# Effects of Impurity Doping on the Luminescence Performance of Mn^4+^-Doped Aluminates with the Magnetoplumbite-Type Structure for Plant Cultivation

**DOI:** 10.3390/ma12010086

**Published:** 2018-12-27

**Authors:** Xiaoshuang Li, Zikun Chen, Bo Wang, Ruizhao Liang, Yongting Li, Lei Kang, Pengfei Liu

**Affiliations:** 1 School of Applied Physics and Materials, Wuyi University Jiangmen, Jiangmen 529020, China; lixiaoshuang12@mails.ucas.ac.cn (X.L.); chenzk1993@163.com (Z.C.); liangliang199912@163.com (R.L.); 13680411460@163.com (Y.L.); kanglei8801@163.com (L.K.); 2 Dongguan Neutron Science Center, Dongguan 523803, China; pfliu@ihep.ac.cn

**Keywords:** LED, phosphor, Mn^4+^, luminescence

## Abstract

Mn^4+^ activated LaMgAl_11_O_19_ (LMA/Mn^4+^) with red emitting phosphor was obtained by sintering under air conditioning. The X-ray diffraction pattern Rietveld refinement results reveal that three six-fold coordinated Al sites are substituted by Mn^4+^ ions. Furthermore, the chemical valence state of manganese in the LMA host was further confirmed through X-ray photoelectron spectroscopy (XPS) and electron paramagnetic resonance (EPR). Photoluminescence emission (PL) and excitation (PLE) spectra of LMA/Mn^4+^ as well as the lifetime were measured, and the 663 nm emission is ascribed to the ^2^E_g_→^4^A_2g_ from the 3*d*^3^ electrons in the [MnO_6_]^8−^ octahedral complex. The emission spectrum matches well with the absorption of phytochrome. Temperature-dependent PL spectra show that the color changes of the phosphor at 420 K are 0.0110 for Δx and −0.0109 for Δy. Moreover, doping Zn^2+^ and Mg^2+^ ions in the host enhances the emission intensity of Mn^4+^ ions. These results highlight the potential of LMA/Mn^4+^ phosphor for a light-emitting diode (LED) plant lamp.

## 1. Introduction

Indoor agriculture has attracted considerable attention because of its relatively stable grow environment without outside interference [1,2]. Studies show that light distribution in blue (400–500 nm) and red (600–690 nm) regions has significant implications for plants as it affects the photosynthetic reaction along with the developmental processes of flowering [3,4,5]. Very recently, phosphor converted light emitting diodes (pc-LEDs) have been recognized as the primary artificial light source for indoor plant growth because of its unique advantages over the traditional gas-discharge lamps, such as simple fabrication technology, being power-economical, low radiant heat output, and the ease of controlling the spectral composition [6,7,8,9,10].

Currently, LED grow light could be produced by the blue-emitting LED, which is prepared though packing of a red phosphor on the GaN chip surface using silicone [11]. Moreover, the plant growth lamps are adjustable according to demand by changing the spectra and luminous efficacy of the phosphor. In particular, the radiation at 650–750 nm wave-bands is necessary for plant growth, thus red phosphor has a major effect on the plant lamps [4,7,12]. It is well known that the transition metal ion Mn^4+^ (3*d*^3^ electronic configuration) presents broad and intense absorption in the near ultraviolet (n-UV) and blue region, resulting from the ^4^A_2_→(^4^T_1_, ^2^T_2_, and ^4^T_2_) spin-allowed transitions, and emits red to near-infrared (NIR) light, arisen by the transition from ^2^E→^4^A_2_ in an octahedral coordination environment [13,14,15,16,17]. Furthermore, the Mn^4+^ ion doped oxide red phosphors perform well as red luminescent materials for LED plant lamps, as demonstrated in Ba_2_TiGe_2_O_8_ [5], La(MgTi)_1/2_O_3_ [18], NaLaMgWO_6_ [7], Li_2_MgZrO_4_ [8], and so on. Besides, LaMgAl_11_O_19_ (LMA) with a magnetoplumbite structure exhibits excellent physical and chemical stability and contains an amount of [AlO_6_] octahedral sites to accept doped Mn^4+^ ions into the LMA lattice [19]. The LaMgAl_11_O_19_ host doped with Mn^2+^ ions has been reported in a great deal of research [20,21,22,23]. However, the related luminescence property of LaMgAl_11_O_19_/Mn^4+^ (LMA/Mn^4+^) phosphor is rarely reported.

In this study, a promising red-emitting Mn^4+^-activated LMA phosphor for plant growth will be reported. The chemical valence state of manganese in the LMA host was investigated by electron paramagnetic resonance (EPR) and X-ray photoelectron spectroscopy (XPS). As expected, the LMA/Mn^4+^ phosphor shows a broad excitation band in the UV-blue region, and a narrow red light emission band peaking at 663 nm, which, in accordance with the absorption band of phytochrome, is observed just under 467 nm. The optimal doping concentration of Mn^4+^ ion is 1 mol %, and the influence of the doping concentration of Mn^4+^ ions on luminescent property is discussed. By introducing impurities to the LMA host, such as Zn^2+^ and Mg^2+^ ions, the luminescent efficiency is significantly improved. All these results demonstrate the great potential of LMA/Mn^4+^ phosphors for application in the agriculture industry as red-emitting luminescent materials.

## 2. Experimental Details

### 2.1. Sample Preparation

Sample LaMgAl_11_O_19_/xMn^4+^ (LMA/xMn^4+^) phosphors were prepared by the solid-state reaction method. The starting raw materials were La_2_O_3_, Al_2_O_3_, MgO, MnCO_3_, and R (R = Li_2_CO_3_, Na_2_CO_3_, MgO, CaO, ZnO, GeO_2_). LaMgAl_11−x−y_O_19_/xMn^4+^/yR (x = 0.02%~5.0%; y = 1% mol) was synthesized by calcination of the mixture of starting materials at 1600 °C for 6 h in an ambient atmosphere. The samples were prepared and then ground in an agate mortar.

### 2.2. Sample Characterization

Structural characterizations were executed by X-ray diffraction (XRD) measurements (X’ Pert PRO, Cu K_α_, λ = 1.5418 Å, PANalytical, Holland). The morphology and grain size of the LMA/0.01Mn^4+^ were investigated using a scanning electron microscope (SEM, Zeiss Sigma500, Jena, Germany). The program Material Studio (MS 5.5, Biovia, San Diego, CA, USA) was used to analyze the crystal structure and atomic position. The photoluminescence (PL) and photoluminescence excitation (PLE) were recorded by Edinburgh Instruments (FLS 980; Livingston, UK) equipped with 450 W xenon lamps as a lighting source. The quantum efficiency (QE) measurements were performed by the spectrophotometer with a barium sulfate coated integrating sphere. The QE, which is defined as the ratio of the total number of photons emitted (*I_em_*) to the number of photons absorbed (*I_abs_*), is expressed as

$$\eta =\frac{I_{em}}{I_{abs}}=\frac{\int L_{S}}{\int E_{R}-\int E_{S}}$$

where *L_S_* is the emission spectrum of the sample, and *E_S_* and *E_R_* are the spectra of the excitation light with and without the sample in the integrating sphere, respectively. The diffuse refection (DR) spectra of the samples were measured by a UV–Vis–NIR spectrophotometer (Lambda 950, Pzserkin Elmer, Canton, MA, USA), using BaSO_4_ as a standard reference. The electron paramagnetic resonance measurement was carried out using a Bruker A300 (Rheinstetten, Germany) with the microwave frequency fixed at 9.8 GHz. The X-ray photoelectron spectroscopy (Thermo ESCALAB 250XI, Waltham, MA, USA) was conducted by Thermo Fisher Scientific. The first-principles calculations for LMA were performed by CASTEP (MS 5.5, Biovia, San Diego, CA, USA) [24], a plane-wave pseudopotential total energy package based on density functional theory (DFT) [25]. The Perdew–Burke–Emzerhoff (PBE) function within the generalized gradient approximation (GGA) form was adopted to describe the exchange-correlation energy. The optimized norm-conserving pseudopotentials in the Kleinman–Bylander [26] form for all the elements were used to model the effective interaction between atom cores and valence electrons. The high kinetic energy cutoff 1000 eV and dense 5 × 1 × 5 Monkhorst–Pack [27] *k*-point meshes in the Brillouin zones were chosen for LMA.

## 3. Results and Discussion

### 3.1. Phase Purity and Crystal Structure Analysis

**Figure 1:**
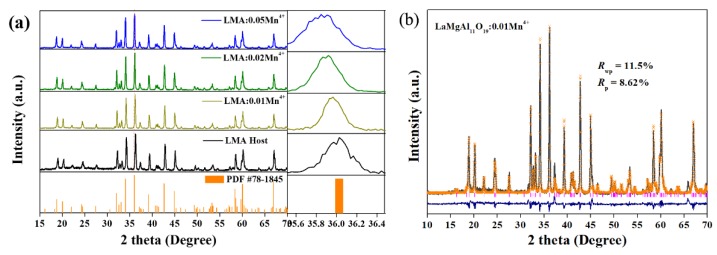
(**a**) X-ray diffraction (XRD) pattern of LaMgAl11O19 (LMA)/xMn^4+^; (**b**) experimental (black solid line) and calculated (yellow crossed symbol) XRD profiles of LMA/0.01Mn^4+^. The difference profile (blue solid line) and Bragg position (vertical line) are also provided.

The XRD patterns of LMA/xMn^4+^ (x = 0, 0.01, 0.02, and 0.05) were conducted and are shown in Figure 1a. It is obvious that all the XRD patterns of the as-prepared samples are consistent with the standard data of LaMgAl_11_O_19_ (PDF #78-1845), indicating that there is not an observable change of the crystal structure with Mn^4+^ ion doping. Then, the influence of the doping concentration of Mn^4+^ ion on the crystal structure was studied by comparing the dominant diffraction peak at 36.16°. Apparently, the diffraction peak gradually shifts to the lower angles with the increased doping concentration of Mn^4+^ ion, which is the result of the bigger ions Mn^4+^ occupying the Al^3+^ ion sites in the LMA host lattice.

To achieve crystallographic data of the prepared samples, LMA/0.01Mn^4+^ was selected as the representative to carry out XRD Rietveld refinement (Figure 1b). The refining adopted the crystallographic data of LaMgAl_11_O_19_ (ICSD #48171) as an initial model and it converged to *R*_wp_ = 11.5%, demonstrating the high reliability of the refined results [28]. Comparing the different profiles between the experimental and calculated ones, we can confirm that the sample has a hexagonal structure belonging to the *P63/mmc* space group. The lattice parameters of *a* = 5.5950(6) Å, *c* = 21.9680(2) Å, *α* = 90°(4), *γ* = 120°(8), and cell volume *V* = 594.86(3) Å^3^ are consistent with reference data, as listed in Table 1.

**Figure 2:**
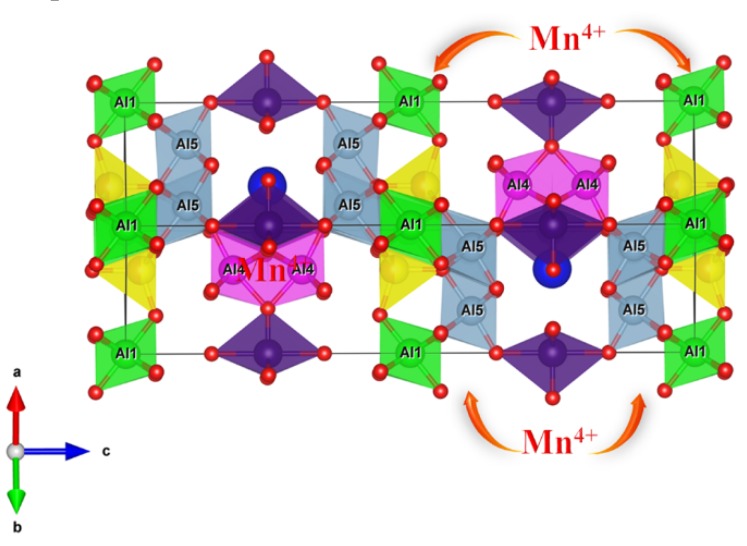
The crystal structure of LaMgAl_11_O_19_ and the octahedral AlO_6_ ligands.

Many previous studies have proven that Mn^4+^ ions in octahedral geometry will produce red emission [11]. Hence, the more [Al(O/F)_6_] octahedral sites the structure has, the greater the capacity it will offer to adopt Mn^4+^ ions, which is beneficial to emit efficient red light. As shown in the LMA structure built based on the refining results (Figure 2), the coordination environments of Al_1−5_ atoms can be observed. It is worth noting that there are three six-fold coordinated Al sites (Al_1_, Al_4_, and Al_5_), which are identical to the coordination situation in other Mn^4+^ ion doped oxide hosts. Correspondingly, the Mn^4+^ ions could substitute for the Al_1_, Al_4_, and Al_5_ sites in the LMA host to form the [MnO_6_]^8−^ octahedral complex.

**Figure 3:**
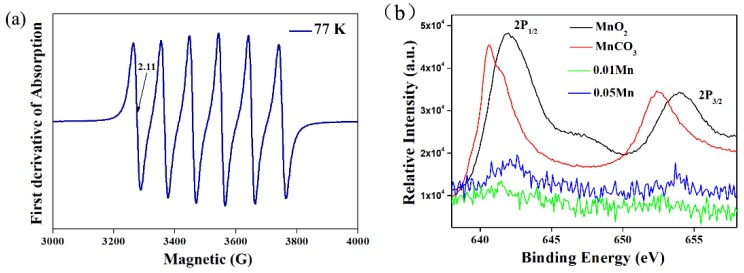
(**a**) Electron paramagnetic resonance (EPR) spectrum of LMA/0.01Mn^4+^ at 77 K; (**b**) the Mn 2*p* X-ray photoelectron spectroscopy (XPS) spectra of MnO_2_, MnCO_3_, and LMA/0.01Mn^4+^ and LMA/0.05Mn^4+^.

In order to assess the manganese (IV) center obtained in the phosphor, the EPR spectrum of LMA/0.01Mn^4+^ at 77 K was conducted. As shown in Figure 3a, the typical hyperfine sextet doubly degeneracy energy levels are observed, indicating the Mn ions possess a strong crystal field. The center of the signals corresponding to the *g* value is 2.11 based on the following equation:

(1) hv=gβH

where *h* is the Planck constant (6.626620 ×10^−27^ erg/s), *ν* is the microwave frequency, *g* is the nondimensional spectral splitting factor (*g* value), and *β* is the Bohr magneton (9.27410×10^−21^ erg/G). The result verifies that the magnetic dipolar transition of Mn^4+^ ions occupies symmetric octahedral sites. Meanwhile, XPS was further used to confirm the chemical valence state of manganese in LMA/Mn^4+^. The XPS spectra of LMA/0.01Mn^4+^, LMA/0.05Mn^4+^, MnCO_3_, and MnO_2_ are displayed in Figure 3b, in which MnCO_3_ and MnO_2_ are used as the reference standards for Mn^2+^ and Mn^4+^ ions, respectively. The peaks of the as-synthesized sample could be assigned to the Mn 2*p*_3/2_ and are close to the peaks of MnO_2_. Meanwhile, the peak intensity increases with the increasing content of Mn as shown in Figure 3b. All these results suggest that the oxidation state of manganese in the LMA host is +4 [29,30].

**Figure 4:**
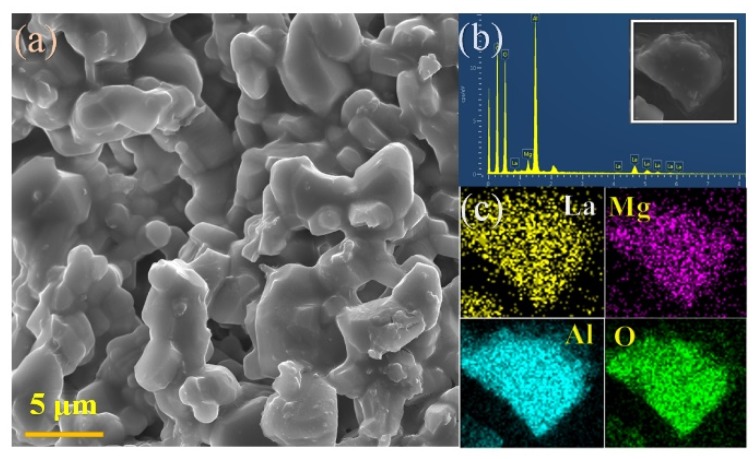
(**a**) Scanning electron microscope (SEM) image of LMA/0.01Mn^4+^; (**b**) EDS spectra of LMA/0.01Mn^4+^; (**c**) SEM and element distribution mapping of the corresponding sample.

Scanning electron microscope (SEM) and energy dispersive X-ray spectroscopy (EDS) were performed to further display the detailed morphological feature and elements of LMA/0.01Mn^4+^ particles. As shown in Figure 4a, the size of the selected particles is about 5–15 μm, illustrating the good crystallization of the as-synthesized sample. The EDS analysis of the phosphors is presented in Figure 4b. All the targeted peaks of the elements (lanthanum (La), magnesium (Mg), aluminum (Al), and oxygen (O)) could be clearly observed. The elemental mapping was carried out to further confirm the uniform distribution of elements. The EDS image shows the presence of La, Al, Mg, and O in the sample, as well as the homogenous distribution of the corresponding elements in the phosphor (Figure 4c).

### 3.2. Electronic Structure Calculations of LMA

**Figure 5:**
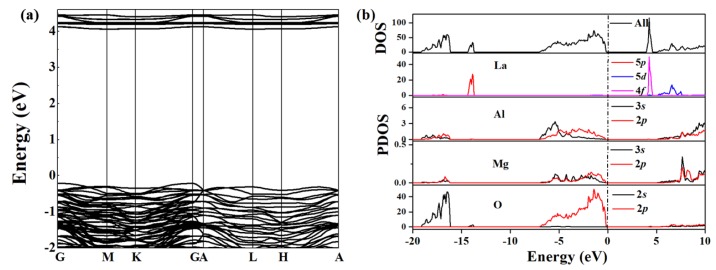
(**a**) Calculated energy band structure of LMA; (**b**) total and partial (La, Al, Mg, and O atoms) density of states (DOS) for LMA. PDOS—partial DOS.

The electronic structure of LaMgAl_11_O_19_ is presented in Figure 5a. The top of the valence band (VB) maximum and the bottom of the conduction band (CB) minimum locate at different *k*-points, revealing that LaMgAl_11_O_19_ is an indirect semiconductor with a band gap of 4.05 eV. The wide band gap demonstrates that the LaMgAl_11_O_19_ is a good luminescent material for accommodating the ^4^A_2g_ and ^2^E_g_ states of Mn^4+^ ion [16]. Additionally, Figure 5b shows the partial density of states (DOS) of La, Mg, Al, and O. Corresponding with the total DOS for LaMgAl_11_O_19_, the VB top is mainly composed of O-2*p*, while La-4*f* states make a significant contribution to the CB top.

### 3.3. Components Luminescent Properties of LMA/Mn^4+^

**Figure 6:**
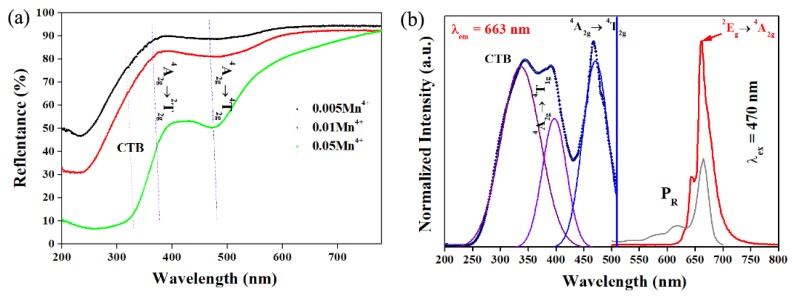
(**a**) The diffuse reflection (DR) spectra of LMA/xMn^4+^ (x = 0.005, 0.01, and 0.05); (**b**) photoluminescence (PL) and PL excitation (PLE) spectra of LMA/0.01Mn^4+^ at room temperature and the absorption spectrum of phytochrome P_R_ (P_R_ is defined as the red light absorbed by the phytochrome).

Figure 6a depicts the diffuse reflection (DR) spectra of LMA with different doping concentrations of Mn^4+^ ion. Similar to the results reported previously, there is a characteristic intense spin-allowed Mn^4+^/^4^A_2g_→^4^T_2g_ transition peaking at ~470 nm and a weak recognizable spin-forbidden ^4^A_2g_→^2^T_1g_ one located at 390 nm [11,16]. However, the charge transfer band is too weak to separate from the DR spectra.

Figure 6b shows the normalized photoluminescence (PL) and PL excitation (PLE) spectra of the LMA/0.01Mn^4+^ at room temperature. Apparently, one prominent red emission band at 663 nm caused by the anti-stokes and stokes transition ^2^E_g_, ^2^T_2g_→^4^A_2g_ of the 3*d*^3^ electrons from Mn^4+^ ions in the [MnO_6_]^8−^ octahedra is observed under excitation of 467 nm [11]. The chromaticity coordinate of the LMA/Mn^4+^ sample in the Commission Internationale de L’Eclairage (CIE) 1931 color spaces is calculated to be (0.725, 0.274), which is beneficial to the long-day plants. The PLE spectrum of the sample comprises two ultraviolet peaks at 345 nm and 390 nm, and one distinguishable blue band centered around 470 nm derived from the inner *d*–*d* transitions of Mn^4+^ ions. The peak locations are the same as the absorption peaks in the DR spectra and the peaks originate from the Mn^4+^–O^2−^ charge-transfer band (CTB) ^4^A_2g_→^4^T_1g_ and spin-allowed transitions ^4^A_2g_→^4^T_2g_ of Mn^4+^ ions, respectively. The internal quantum efficiency of LMA/Mn^4+^ is measured to be 38.5% for the excitation wavelength of 465 nm, as demonstrated in Figure S1. The value is comparable to the one reported by Peng et al. [13]. These spectral features agree well with other previous studies about Mn^4+^-doped aluminates, suggesting that the Mn^4+^ ions had been successfully incorporated into the LMA host. The Tanabe–Sugano energy-level diagram illustrates the dependence of energy levels of 3*d*^3^ on the parameters of Dq, B, and C, in which Dq is a parameter that characterizes the strength of the octahedral crystal field, while B and C are Racah parameters. From the supplementary Equations (1)–(4), the values of Dq, B, and C in the LMA/Mn^4+^ are then determined to be 2150 cm^−1^, 321 cm^−1^, and 4076 cm^−1^, respectively.

**Figure 7:**
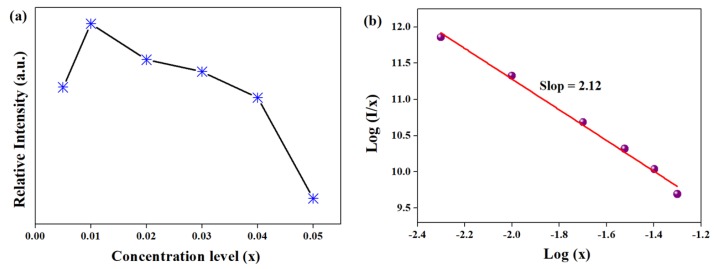
(**a**) The PL integrated intensities with Mn^4+^ concentration, (**b**) as well as dependence of log(I/x) on log(x).

The doping concentration of Mn^4+^ ion dependence of the PL integrated intensity is shown in Figure 7a. Typically, the luminescence intensity increases gradually at first with the increase of the doping concentration of Mn^4+^ ion, then approaches a maximum value at 0.01, and finally decreases when the doping concentration of Mn^4+^ ion is slightly higher than 0.01. The exchange interaction or multipole–multipole interaction within the nearest Mn^4+^ ions are ascribed to the concentration quenching phenomenon. To clarify this point, it is necessary to calculate the critical transfer distance (*R*_c_) among the Mn^4+^ ions. Upon the Blasse mechanism [31], *R*_c_ is evaluated via the following equation:

(2) $$R_{C}\approx 2{\left(\frac{3V}{4\pi x_{c}Z}\right)}^{\frac{1}{3}}$$

In this work, *V* = 594.86 Å^3^, N = 28, *x*_c_ = 0.01, and the critical *R*_c_ of Mn^4+^ ion in LMA is calculated to be ~15.9 Å. The estimated *R*_c_ value is bigger than 5 Å, hence it is inferred that an exchange interaction may not be the main possible approach.

Thus, the concentration quenching mainly relies on the electric multipolar interaction, which is confirmed by Dexter’s theory [32]. The type of interaction between Mn^4+^ ions can be expressed by the following equation:

(3) $$\frac{I}{x}=K{\left[1+\beta {\left(x\right)}^{\frac{\theta }{3}}\right]}^{-1}$$

where *x* is the activator concentration; *K* and *β* are constants; and *θ* is an indication of the electric multipolar character in which *θ* = 6, 8, 10 corresponds to dipole–dipole, dipole–quadrupole and quadrupole–quadrupole, respectively. Figure 7b depicts the dependence of log(x) and log(I/x) for the LMA/Mn^4+^ phosphors. The linear slope is calculated to be ~2.12 and the value of *θ* is determined as approximately 6. Consequently, the quenching mechanism is a dipole–dipole interaction in LMA/Mn^4+^.

**Figure 8:**
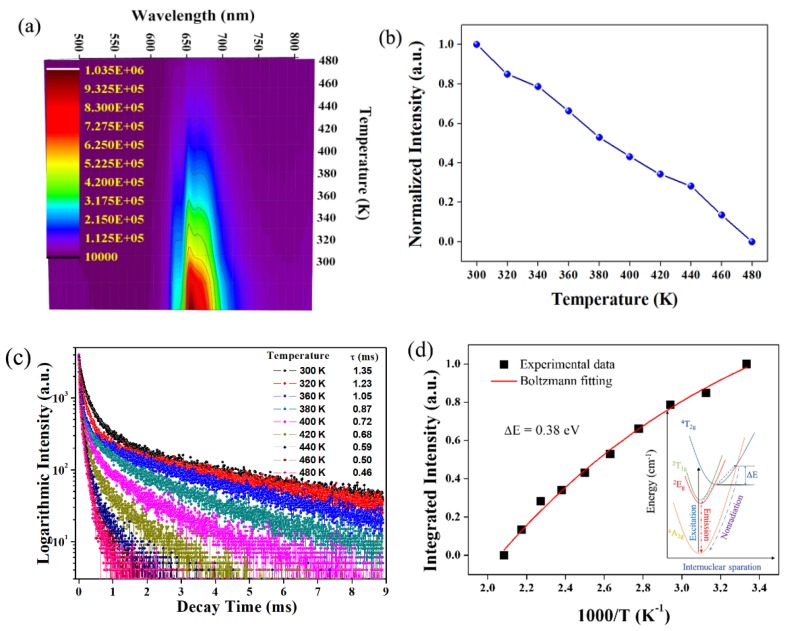
(**a**–**c**) Temperature-dependent emission spectra, integrated intensity, and lifetime of LMA/0.01Mn^4+^, respectively; (**d**) configurational coordinate diagram of Mn^4+^ in the LMA host for the possible thermal quenching process.

Generally, as a red color converter in blue-chips, the thermal stability of the sample is a significant parameter in fundamental research, because the chip temperature usually undergoes a high temperature (> 423K), which would degrade emission intensity and color quality. Figure 8a gives the temperature-dependent emission spectra of LMA/0.01Mn^4+^ spanning from 300 K to 480 K under excitation of 467 nm. Obviously, the emission intensity of the as-prepared sample decreases monotonically with the increase of temperature. However, there is no distinct peak position shift over 380 K. The integrated emission intensity is presented in Figure 8b. When the sample is heated to 420 K, the integrated intensity retains only 36% of the emission intensity at 300 K. The thermal quenching behavior may be affected by the oscillator strength and the distributed killer centers, which could strengthen the nonradiative energy relaxation. Despite an obvious drastic emission loss, the chromaticity coordinate variations at 420 K are 0.0110 and −0.0109, respectively, well satisfying the requirement in LED applications [33]. Meanwhile, the temperature-dependent PL decay curves of LMA/0.01Mn^4+^ at 663 nm were further measured. As shown in Figure 8c, the decay lifetimes seem to be multiexponential. We found that there are three six-fold coordinated Al sites (Al_1_, Al_4_, and Al_5_) in the structure of LMA. When Mn^4+^ ions substitute for the Al_1_, Al_4_, and Al_5_ sites in the LMA host, three [MnO_6_]^8−^ octahedral complex luminescence center formed, which would contribute to the decay time with multiexponential function. Meanwhile, the doping concentration of Mn^4+^ ions also affect the decay process via energy transfer. The distance between the activated centers is 2.658 to 5.59Å, hence the energy transfer would lead to the deviation from linear of the decay curves. Moreover, the decay lifetimes also strongly rely on the temperature. The lifetimes are calculated according to the equation below:

(4) $$\tau =\frac{\int_{0}^{\infty }I\left(t\right)tdt}{\int_{0}^{\infty }I\left(t\right)dt}$$

where *τ* is the decay time and *I*(t) is the luminescence intensity at time t. Based on Equation (4), the average lifetime of LMA/0.01Mn^4+^ decreases gradually from 1.35 ms at 300 K to 0.46 ms at 480 K, which is consistent with the change rule of the emission intensity. The decay of Mn^4+^ in the matrix becomes faster and faster, owing to the increased nonradiative energy migration among Mn^4+^ ions at a higher temperature.

The activation energy (ΔE) for thermal quenching can be determined by the following equation [34]:

(5) $$I_{T}=I_{0}/\left[1+c\exp\left(-∆E/kT\right)\right]$$

where *I_0_* and *I_T_* stand for the initial emission intensity and the luminescence intensity at temperature *T*, respectively; c is a constant for a designated host; and k is the Boltzmann constant. Thus, the activation energy of the LMA/Mn^4+^ phosphor is calculated to be 0.38 eV. The thermal quenching mechanism can be explained by the configurational coordinate diagram, as shown in Figure 8d.

**Figure 9:**
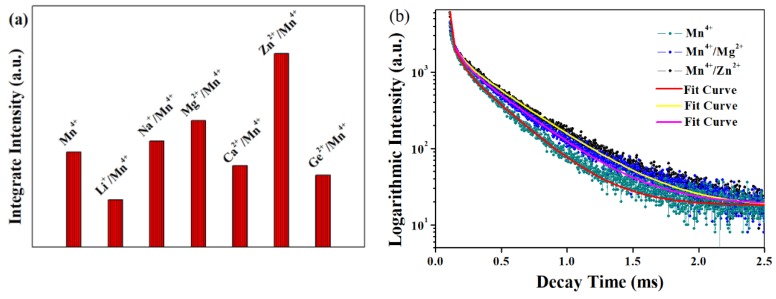
(**a**) Integrated intensity for the Mn^4+^ (0.01 mole)/LMA and Mn^4+^/M/LMA (M = Li^+^, Na^+^, Mg^2+^, Ca^2+^, Zn^2+^, Ge^4+^) phosphors; (**b**) decay curves of Mn^4+^ with Mg^2+^, Ca^2+^, and Zn^2+^ doping as well as without doping.

In a further experiment, the influence of M (M = Li^+^, Na^+^, Mg^2+^, Ca^2+^, Zn^2+^, Ge^4+^) ion on the emission intensity of Mn^4+^/LMA was studied (Figure S2). The ionic radii of these M ions are 0.590 Å for Li^+^ (CN = 4), 0.99 Å for Na^+^ (CN = 4), 0.720 Å for Mg^2+^ (CN = 6), 1.00 Å for Ca^2+^ (CN = 6), 0.60 Å for Zn^2+^ (CN = 4), and 0.530 Å for Ge^4+^ (CN = 6), respectively [35]. When the impurity ions were co-doped into LMA/Mn^4+^, the characteristic peaks in the emission spectra were not changed. The integrated PL intensities of the samples into which the impurity ions were incorporated are presented in Figure 9a. Therein, Mg^2+^ and Zn^2+^ ions have the ability to enhance luminescence of LMA/0.01Mn^4+^, Na^+^ and Ca^2+^ ions have no obvious impact on Mn^4+^ ion luminescence, and the Li^+^/Ge^4+^ doped LMA/0.01Mn^4+^ phosphors show weaker luminescence intensities under excitation of the same blue light. Specifically, the ratio of the emission yields of LMA/0.01Mn^4+^ phosphors with and without Zn^2+^ ion doping are measured to be 2.04, in favor of the conclusion that the luminescence intensity of LMA/Mn^4+^ can be greatly strengthened via impurity doping. Moreover, the effect of co-dopants for improving Mn^4+^ luminescence follows the order of Zn^2+^ > Mg^2+^ > Na^+^. As discussed in Figure 8c, the substitution of Mn for the Al_1_, Al_4_, and Al_5_ sites in the LMA host is attributed to the multiexponential function; the co-doped model also presents similar decay curves. Similarly, the decay curves were tested to verify this phenomenon. Figure 9b shows the lifetime of Mn^4+^ ion in phosphor with co-doping Zn^2+^ and Mg^2+^ ions, and the result suggests that the lifetime becomes longer. The introduction of Mg^2+^/Zn^2+^ ions increases the PL intensity of Mn^4+^ ions, which is attributed to the replacement of Mn^4+^–Mn^4+^ pairs by Mg^2−^–Mn^4+^ and/or Zn^2+^–Mn^4+^ ion pairs. Hence, the nonradiative depopulation of the ^2^E_g_ state is decreased because the energy migration between the Mn^4+^–Mn^4+^ ion pairs is faster than Mg^2−^–Mn^4+^ and/or Zn^2+^–Mn^4+^ ion pairs, and thus the probability of the energy terminating at a killer site of the Mn^4+^–Mn^4+^ ion pairs is greater. Moreover, the formation of Mg^2+^–Mn^4+^/Zn^2+^–Mn^4+^ pairs along with the impurities of Mg^2+^/Zn^2+^ ions not only lengthen the emission lifetimes of the co-doped samples compared with that of the LMA/0.01Mn^4+^ sample, but also lead to the decrease in the nonradiative rate. [13,16].

## 4. Conclusions

In summary, a red emitting phosphor LMA/Mn^4+^ for an LED plant growth lamp was synthesized by the solid state reaction method and sintering at 1600 °C in the air directly. The crystal structure of LMA/0.01Mn^4+^ was studied by the XRD Rietveld refinements. XPS and EPR spectra demonstrate that the chemical valence of manganese is +4. The LMA/Mn^4+^ phosphor exhibits a red emission peaking at 663 nm, which is attributed to the ^2^E→^4^A_2_ transition of Mn^4+^ ion in the [MnO_6_]^8−^ octahedral environment, and the QE is 35.8% under the blue-light excitation. The optimal doping concentration of Mn^4+^ ion is 0.01 mole and the quenching mechanism is a dipole/dipole interaction. Moreover, the chromaticity coordinate variations at 420 K are 0.0110 and −0.0109, respectively. Additionally, the luminescence of LMA/Mn^4+^ could be greatly enhanced via impurity doping Zn^2+^ or Mg^2+^ ions. These properties make LMA/Mn^4+^ a promising red emitting phosphor for plant growth LEDs.

## Figures and Tables

**Table 1:** Crystallographic data and refinement parameters of the LMA/0.01Mn^4+^.

Atom	x	y	z	Occ	U	Coordination Number
La	0.33333	0.66667	0.75000	1	0.00911	
Mg	0.33333	0.66667	0.02720	0.5	0.00240	4
Al1	0.00000	0.00000	0.00000	1	0.00190	6
Al2	0.00000	0.00000	0.25000	1	0.02305	5
Al3	0.33333	0.66667	0.02720	0.5	0.00240	4
Al4	0.33333	0.66667	0.18950	1	0.00228	6
Al5	0.16740	0.33480	0.89200	1	0.00304	6
O1	0.00000	0.00000	0.15100	1	0.00278	
O2	0.33333	0.66667	0.94200	1	0.00329	
O3	0.18000	0.36000	0.25000	1	0.00709	
O4	0.15200	0.30400	0.05300	1	0.00455	
O5	0.50500	0.010000	0.15100	1	0.00380	
Symmetry:	hexagonal		Space group:		*P63/mmc*	
Lattice parameters: *a* = *b* = 5.5950 Å; *c* = 21.9680 Å; *α* = β = 90°; *γ* = 120°; V = 594.86 Å^3^						

## Supplementary Materials

Supplementary materials can be found at http://www.mdpi.com/1996-1944/12/1/86/s1. Figure S1: The quantum efficiency of the LMA:0.01Mn^4+^ phosphor, Figure S2: PL spectra of LMA:0.01Mn^4+^, 0.01M^+^ powder.
