# Supplementary material for: Effects of Impurity Doping on the Luminescence Performance of Mn4+-Doped Aluminates with the Magnetoplumbite-Type Structure for Plant Cultivation
Source: Materials (Basel). 2018 Dec 27;12(1):86. doi: 10.3390/ma12010086 (PMC6337654; doi:10.3390/ma12010086)
Supplement: Supplementary file 1 [file materials-12-00086-s001.pdf]

materials

Article

Effects of impurity doping on the luminescence performance of Mn^{4+}-doped aluminates with the magnetoplumbite-type structure for plant cultivation

Xiaoshuang Li [1], Zikun Chen [1], Bo Wang [1,*], Ruizhao Liang [1], Yongting Li [1], Lei Kang [1] and Pengfei Liu [2]

[1] School of Applied Physics and Materials, Wuyi University Jiangmen, Guangdong 529020, P.R. China; wangbo312@mails.ucas.ac.cn (B. Wang)
[2] Dongguan Neutron Science Center, Dongguan 523803, PR China
* Correspondence: wangbo312@mails.ucas.ac.cn (B. Wang)

Supplementary Materials

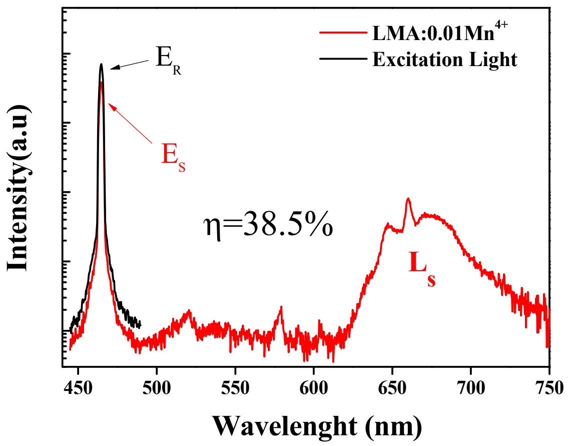

Figure. S1. The quantum efficiency of the LMA:0.01Mn^{4+} phosphor.

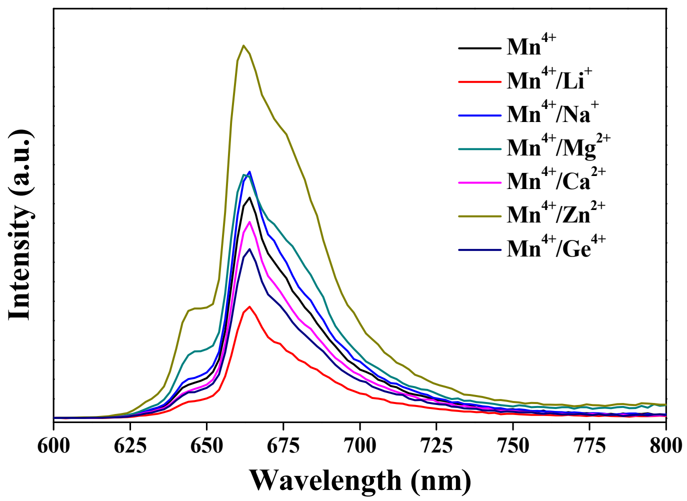

Figure. S2. PL spectra of LMA:0.01Mn^{4+}, 0.01M^{+} powder.

Calculation of crystal field parameters of Dq, B, and C

The values of Dq, B, and C can be calculated based on experimentally determined energy levels using the following equations:[1,2]

$$Dq = E({}^4T_{2g} - {}^4A_{2g})/10 \tag{1}$$

$$\frac{Dq}{B} = \frac{15(x-8)}{(x^2 - 10x)} \tag{2}$$

$$x = \frac{E({}^4A_{2g} \rightarrow {}^4T_{1g}) - E({}^4A_{2g} \rightarrow {}^4T_{2g})}{Dq} \tag{3}$$

$$E({}^2E_g - {}^4A_{2g})/B = 3.05C/B + 7.9 - 1.8B/Dq \tag{4}$$

where ΔE is the energy difference between ${}^4T_{2g}$ and ${}^4T_{1g}$. The energy levels of ${}^4T_{2g}$, ${}^4T_{1g}$, and ${}^2E_g$ were determined at 21505, 25316, and 15083 cm^{-1}. From equations (1)-(4), the values of Dq, B, and C in the CMA: Mn^{4+} are then determined to be 2150 cm^{-1}, 321 cm^{-1}, 4076 cm^{-1}, respectively.

Reference

1. Henderson, B.; Imbusch, G.F. Optical spectroscopy of inorganic solids. Oxford University Press, **1989**.
2. Reisfeld, M.J.; Natwiyoff, N.A.; Asprey, L.B. The electronic spectrum of cesium hexafluoromanganese (IV). *J. Mol. Spectrosc.* **1971**, 39, 8–20.

© 2018 by the authors. Submitted for possible open access publication under the terms and conditions of the Creative Commons Attribution (CC BY) license (http://creativecommons.org/licenses/by/4.0/).
